# Dissociable psychosocial profiles of adolescent substance users

**DOI:** 10.1371/journal.pone.0202498

**Published:** 2018-08-30

**Authors:** Amanda Fitzgerald, Naoise Mac Giollabhui, Louise Dolphin, Robert Whelan, Barbara Dooley

**Affiliations:** 1 UCD School of Psychology, University College Dublin, Newman Building, Belfield, Dublin, Ireland; 2 Department of Psychology, Temple University, Philadelphia, PA, United States; 3 Trinity Institute of Neurosciences (TCIN), School of Psychology, Trinity College Dublin, College Green, Dublin, Ireland; Universidade Federal de Sao Paulo, BRAZIL

## Abstract

**Objective:**

Alcohol, tobacco and cannabis use in adolescence is associated with adverse outcomes. Characterizing adolescent substance misusers, however, is difficult due to the wide range of risk and protective factors linked to substance use. The aim of the present study was to examine the role of the Individual, Family, School, Peer, and Social Environment on alcohol (lifetime and risky), tobacco (risky only), and cannabis use (lifetime and riskiness).

**Method:**

Data were analyzed from a national sample of 5,680 adolescents, capturing substance use behavior alongside risk and protective factors across Individual, Family, School, Peer and Social domains. We applied a sophisticated machine learning classifier to develop models of alcohol, tobacco and cannabis initiation and misuse.

**Results:**

We found highly accurate (area under curve of receiver-operator-characteristic for out-of-sample performance was > .88) and replicable (over multiple iterations and in comparison with permuted outcomes) dissociable psychosocial profiles of alcohol, tobacco and cannabis use. Alongside common predictors (peer relations and externalizing behavior), dissociable risk and resilience factors were observed. Adolescent profiles of alcohol use were distinguished by the contribution of multiple domains. In contrast, tobacco use was characterized by a small number of individual variables, including female gender and poor perceived academic position. Cannabis use was differentiated by the distinct contribution of Individual risk factors, in particular male gender and feelings of anger. Differential associations were also evident, with the strength and direction of association differing substantially across substances.

**Conclusion:**

This study indicates that the relationship between the environment and substance use is more complex than previously thought.

## Introduction

Use of alcohol, tobacco and cannabis (ATC) in adolescence is a known risk factor for concurrent health issues and increased likelihood of adult dependence [1]. Despite these risks, lifetime and recency prevalence estimates for ATC remain high in adolescence [2]. Higher frequency use is associated with poorer outcomes and the use of multiple substances (i.e. polysubstance use) is common [3,4]. Identifying risk (R) and protective (P) factors for adolescent substance initiation and misuse is challenging. There is a growing realization that human substance use is complex, with many diverse, individually weak, factors contributing to the profile of adolescent substance misusers [5].

Adopting an ecological framework approach [6,7] conceptualizing risk and protective factors at the level of the Individual, Family, School, Peer, and Social Environment, is conducive to the construction of complex models of substance use [8]. Individual factors include age [7], gender [9], ethnicity [4], victimization from bullying [10], mental distress [11], self-esteem [12], and coping style [13]. Factors across other ecological domains include family conflict, single parent families, academic failure [14,15], parent and school attachment [16,17], romantic relationships [5], romantic breakups [18], neighbourhood characteristics [19], and getting in trouble with the police [20]. However, risk and protective factors differentially affect ATC use [21]. For example, socio-economic status is a risk factor for cigarette smoking but not for alcohol or cannabis use [22]; family and community protective factors are associated with lifetime cigarette use but not alcohol or cannabis use [7]. Thus, dissociating risk and protective factors for alcohol, tobacco and cannabis is important, rather than solely focusing on single underlying vulnerabilities or considering substance use as a unitary phenotype [23,24].

Previous research has examined multiple risk and protective factors associated with a single substance use phenotype [25,26]. Other research considering multiple ATC phenotypes has typically contained limited factors (e.g., gender and age) [27] or employed clinical rather than community-based samples [28]. Significant methodological impediments have prevented researchers from assaying a wide range of factors over multiple substance use phenotypes. First, large sample sizes are required to detect the small effect sizes that pertain to psychological research, in particular where absolute rates of substance misuse (especially cannabis) are low in young adolescence [29]. Second, the use of stochastic data models may not be optimal for identifying the most relevant features, given intercorrelation among individual features and the dimensionality of the feature set [7,21].

We use a large sample (n = 6,062) from the *My World Survey* [30] to investigate risk and protective factors for alcohol, tobacco and cannabis use across Individual, Family, School, Peer, and Social domains, while controlling for the use of other substances [31]. The objective of this study was to identify the profile of adolescent substance misusers across multiple domains including the Individual, Family, School, Peer, and Social domains. A polysubstance use domain was included in analyses to identify risk and protective factors for specific substances, rather than substance use generally (detailed in Methods below).

We applied regularized regression via the Elastic Net [31], which, owing to its combination of ridge regression [32] and the lasso [33], is a method that both deals with data dimensionality and performs feature selection. Thus, we generated a multivariable predictive model, including both continuous and categorical features. Notably, we report out-of-sample performance using 10-fold cross validation. The validity of the machine learning method was tested by application of the same regression method to data in which the substance use outcomes were randomly re-assigned. Unique and shared variance was quantified by selective removal of domains and testing of domains in isolation.

## Methods

A detailed report of the study design, measures and analyses is provided in S1 File.

### Study design and sample

The My World Survey (MWS) is a cross-sectional community survey of risk and protective factors of mental health. The MWS-Second Level (MWS-SL) study recruited adolescents, aged 12–19 years, from a representative sample of the 732 second-level schools in Ireland. A sample of 10% of second-level schools was set (n = 72), with a proposed sample of approximately 80 students per school, yielding a projected sample of nearly 6,000. To achieve the projected school sample size, 171 randomly selected schools were approached thus the school response rate was 42%. The sampled schools did not depart from the national distribution of schools in terms of gender distribution, disadvantaged status, and proportion of schools per healthcare district. The final sample included 6,062 second-level students from 72 randomly selected schools aged 12–19 years (M = 14.94, SD = 1.63), 51% female (n = 3,101).

### Procedures

Data were collected between February and October 2011. University College Dublin Human Research Ethics Committee approved the study protocol. Informed consent was obtained from participants (verbal consent) and their parents/guardians (written consent), with a participant response rate of 45%. Data were collected during school hours in a classroom setting. Parents were not present during the survey and were not provided with participant’s responses. Full details of the procedures employed by the MWS-SL study including details on ethics, recruitment, and standardized protocol for survey administration in schools are available elsewhere [30].

### Measures

Risk and protective factors were assessed using psychometrically validated self-report measures along with single-item indicators, which were clustered into the following domains: Individual, Family, Peer, School, Social Environment and Polysubstance (see Table 1). The domain of ‘Polysubstance’ was included in all analyses to measure ‘nuisance’ variables in analyses and thereby account for variability in the dependent variable that reflects a general tendency to use substances, rather than a single substance specifically. Thus, in Model A, where the dependent variable is ‘Lifetime Alcohol Use’, two substance use variables were included in the model, namely whether participants had used tobacco or cannabis over the previous month. In the case of tobacco, the use of alcohol and cannabis over the prior month was assessed and in the case of cannabis, alcohol and tobacco use over the prior month was assessed.

**Table 1:** Characteristics of the study population & MWS measures.

Domain	Construct	Summary Statistics	Domain	Construct	Summary Statistics
**Individual**	Gender (% Female)	51.70%	**Family**	MSPSS Perceived Family Support (M/SD)	20.5(5.77)
	School Year (M/SD)	3.21(1.67)		READ–Family Cohesion (M/SD)	22.97(4.8)
	Ethnic Minority (% Caucasian)	95.40%		Enjoy Family Life (% Sometimes/Yes)	28.7%/ 68.1%
	Seen Mental Health Professional (% Yes)	11.50%	**School**	Additional Teaching Support in School (% Yes)	9.70%
	APSS-3 Psychotic Symptoms (M/SD)	.655(.858)		Perceived Academic Position	67.3%/5.2%
	DASS-21 Depression (M/SD)	7.58(8.64)		Disadvantaged School (% Not Disadvantaged)	76.9%
	DASS-21 Anxiety (M/SD)	6.66(7.37)		Mixed School (% Mixed)	66.10%
	DASS-21 Stress (M/SD)	9.11(8.30)		Hemingway MAC- School Connectedness (M/SD)	20.38 (4.6)
	CSI-15 Avoidance Coping (M/SD)	15.7(6.03)		Hemingway MAC- Teacher Connectedness (M/SD)	21(5.03)
	CSI-15 Planning Coping (M/SD)	16.2(5.56)	**Peer**	Experienced Breakup (% Yes, > 1 year ago/ < 1 year ago)	27.9%/31.1%
	CSI-15 Support Coping (M/SD)	14.6(5.38)		Have Romantic Partner (% Yes)	29.50%
	Anger (%Sometimes/ % Yes)	43.8%/10.5%		MSPSS Perceived Peer Support (M/SD)	21.39(5.76)
	Body Dissatisfaction (M/SD)	3.22(1.19)		Hemingway MAC- Peer Connectedness (M/SD)	22.32(3.84)
	BAS-7 Acting Out Behavior (M/SD)	11.74(4.16)	**Social** **Environment**	Safe Neighbourhood	4.35(.862)
	BMSLSS Satisfaction with Life (M/SD)	32.18(6.95)		Live in Urban area	38.70%
	LOT-R Optimism (M/SD)	13.8(4.7)		Exp. Racism (% Yes)	10.90%
	READ–Social Competence (M/SD)	19.14(3.51)		Exp. Bullying (% Yes)	44.90%
	RSE Self-esteem (M/SD)	28.54(5.68)		Trouble with Police (% Yes)	13.50%
	Maternal Employment (% Employed)	63.90%		Informal help seeking	5.19 (1.33)
**Family**	Paternal Employment (% Employed)	83.30%		One Good Adult (% Yes)	3.88 (1.27)
	Maternal Education (% Education<Leaving Certificate)	12.50%		Experienced Bereavement? (% Yes)	28.70%
	Paternal Education	29.30%	**Polysubstance/Outcome**	Lifetime Alcohol (% Yes; Ever Used Alcohol)	51.40%
	Mother Stay at Home Versus Other (% Stay at Home)	30.10%		Alcohol Risk (% Yes; ≥ 5 on AUDIT)	32.10%
	No. Children in household (% 3-5/ %6+)	27.1%/6.7%		Tobacco Risk (% Yes; Used Tobacco in Last Month)	23.50%
	Parental Mental Health Problems (% Yes)	12.10%		Lifetime Cannabis (% Yes; Ever Used Cannabis)	12.30%
	Intact Family (% Intact)	81.10%		Cannabis Risk (% Yes; Used Cannabis in Last Month)	8.20%

#### Dependent variables

Alcohol: Alcohol use was assessed using the Alcohol Use Disorders Identification Test (AUDIT) [34], Cronbach’s alpha in this study was .82. Lifetime use of alcohol was assessed with question one from the AUDIT ‘How often do you drink alcohol? (response alternatives: (i) never, (ii) less than monthly, (iii) monthly, (iv) weekly, (v) daily or almost daily). Participants were grouped according to their alcohol use: (i) those who had never used alcohol versus (ii) those who had used alcohol. Risky alcohol use was measured using a cutoff score of ≥ 5 on the total AUDIT score, where a cutoff at 5 or above indicated risky alcohol use [35].

Tobacco: Tobacco use over the past month, identified as risky tobacco use, was assessed with the item ‘Think back over the last month. How many times over the past month have you smoked cigarettes? (response alternatives: (i) never, (ii) once or twice, (iii) 3 or 4 times, (iv) pretty often, and (v) almost every day). Participants were grouped according to their cigarette use: (i) those who never smoked cigarettes in the past month versus (ii) those who used cigarettes over the past month.

Cannabis: Cannabis use was assessed by two items. Lifetime use of cannabis was assessed with a single item “Have you ever smoked cannabis?” (response alternatives: (i) no, (ii) yes). Cannabis use over the past month (risky cannabis use) was assessed with the item “Thinking back over the last month, how many times have you smoked hash/cannabis?” (response alternatives: (i) never, (ii) once or twice, (iii) 3 or 4 times, (iv) pretty often, and (v) almost every day). Participants were grouped according to their cannabis use: (i) those who never used cannabis in the past month, versus (ii) those who used cannabis during the past month.

#### Independent measures

Information on independent measures used is provided in **S1 File.**

### Machine learning procedure

We conducted logistic regression with Elastic Net regularization [31], which allows relevant but correlated coefficients to coexist in a sparse model fit. Elastic Net regularization imposes a hybrid of both L1- and L2-norm penalties (i.e., penalties on the absolute (L1 norm) and squared values of the β weights (L2 norm)). This allows relevant but correlated coefficients to coexist in a sparse model fit, by doing automatic variable selection and continuous shrinkage simultaneously, and selects or rejects groups of correlated variables. Least absolute shrinkage and selection operator (LASSO) and ridge regression are special cases of the Elastic Net. The model selected using logistic regression with Elastic Net regularization represents optimal classification of cases using the minimal number of predictors.

All predictor data were first feature scaled (z-score transformed). Briefly, to implement cross-validation, the data were randomly split into 10 groups. A model was then generated based on 9 training groups, and then applied to the remaining independent testing group. Each group served as the testing group once, resulting in 10 different models, and predictions for every subject based on independent data. Nested cross-validation involved subdividing the 9 training groups (i.e., 90% of the sample) into a further 10 groups (‘inner’ folds). Within these 10 inner folds, 9 were utilized for training a model over a range of 30 alpha (.01–1) and 30 lambda (.0001–1) values. This generated a resulting model fit on the inner fold test set for each possible combination of alpha and lambda. The mean fit over all 10 inner folds for each combination of alpha and lambda was then calculated and then used to determine the optimal parameters for the outer fold.

Model performance was assessed using the area under the curve of the receiver operator characteristic (AROC) score [36] and the harmonic mean of precision and recall (F1 score) [37]. AROC is a metric that indicates a model’s accuracy in correctly classifying a given binary outcome (e.g, risky alcohol use) by plotting the true positive rate (i.e., the likelihood of correctly identifying a case) by the false positive rate (the likelihood of incorrectly identifying a case) at various threshold settings. AROC model performance can be categorized as: excellent (>0.9), very good (0.8–0.9), good (0.7–0.8), average (0.6–0.7) or poor (<0.6). However, as the base rate declines, the AROC becomes less reliable because high scores are driven by high false positive rates (i.e., correctly classifying true negative cases), rather than high true positive rates (correctly classifying true positive cases). Consequently, when base rates are low (e.g., if 90% of individuals do not engage in a given behavior), the F1 score is a useful compliment. The F1 score represent the harmonic average of precision (proportion of positive cases identified from total number of positive cases) and sensitivity (proportion of positive cases identified from all true positive cases and false negative cases); there are no established cut-offs for the F1 score, however 1 represents perfect sensitivity and specificity and 0 represents no sensitivity or specificity. Since both of these metrics are fundamentally based on rates of true positives, false positives, true negatives, and false negatives, these raw data are also presented in a confusion table.

#### Data processing

The final sample who completed the My World Survey was 6,062 students. Observations in which participants responded to fewer than 50% of survey questions were deleted (n = 73), leaving a sample of 5,989. Additionally, observations in which data were missing for any of the three outcome variables were deleted (n = 309), leaving a sample of 5,680 for analyses. Following the exclusion of observations missing data on the dependent variables and those missing more than 50% of data, multiple imputation was conducted on the remaining data using SPSS (version 22). Multiple imputation was conducted since the machine learning classifier implemented requires complete data. The mean percentage of missing case per item across all measures used in analyses was 2.45% (SD = .02). Missing data analyses compared the 309 participants with missing data on a dependent variable with the retained sample. Independent sample t-tests indicated that the subsample retained in the study differed significantly for age, with the retained sample being significantly older by .98 years, t(341) = 14.29, p < .001. Chi-squared analyses indicate that the retained and analytic sample differed significantly by sex, ***χ***^**2**^(1) = 4.58, p = .03 and ethnicity, ***χ***^**2**^(4) = 2.548, p = .12, but did not differ according to the disadvantaged status of the school, ***χ***^**2**^(1) = 2.55, p = .12, or maternal education, ***χ***^**2**^(4) = 2.03, p = .12. Examination of residuals for gender and ethnicity (white versus non-white) did not reveal substantial differences across cells for gender (Standardized residuals less than 2) however a greater number of participants identifying as ‘black’ (Standardized residuals equals 2.8) were excluded from analyses than expected. Although statistically significant differences were observed, there is no clear evidence that substantial differences between the excluded and analytic samples are observable that may bias results.

## Results

Characteristics of the population along with data on frequency of substance use are presented in Table 1. Five models were generated predicting: lifetime alcohol use (Model A); risky alcohol use (Model B); risky tobacco use (Model C); lifetime cannabis use (Model D) and risky cannabis use (Model E). Each of these models represent the optimal model for the classification of substance use based on available data when all domains of predictors were available for selection. The machine learning algorithm dropped variables from the model that did not improve the accuracy of classification. When evaluating the models generated, a number of pieces of information were considered. The central question is whether Models A–E perform well in correctly classifying types of substance use in adolescents? Overall model fit was assessed using the AROC and the F1 score. Additionally, to ensure that the models generated produced meaningful predictive models, the machine learning algorithm was also run on data where the outcomes were shuffled at random. Should accurate models be generated, this would indicate that the machine learning classifier algorithm is not producing meaningful predictive models. Second, we were interested in assessing the contribution that each domain of predictors towards the overall predictive power of the model. Consequently, Models A—E were run after (i) consecutively removing a domain of predictors and (ii) only using a single domain of predictors. This step allows quantification of the contribution that each domain of predictors offers to Models A–E. Where indices of overall model fit do not decline, this would indicate that a domain of predictors do not substantially contribute to classification of substance use, even if they provide minor, incremental predictive power to the model. Finally, the significance of each individual predictor is considered in the Models A–E.

### Model performance

Model performance was assessed by two performance metrics: AROC and by the F1 score- where groups were significantly imbalanced with respect to sample size more attention is given to the F1 score.

The lifetime alcohol model returned an AROC of 0.8924 (95% CI = 0.8923–0.8925) and an F1 score of .81. At the optimum point of the ROC curve, 81% of those who had used alcohol and 81% of those who had not used alcohol were classified correctly with a probability significantly better than chance (*p* ≈ 0). The AROC for the lifetime alcohol model where outcomes were shuffled was at chance 0.496 (95% CI = 0.493–0.500). The risky alcohol use model reported an AROC of 0.9051 (95% CI = 0.9050–0.9052) and an F1 score of .7688; 68% of those above the cut-off and 91% of those below were classified correctly (*p* = 8.11 X **10**^**−160**^). Shuffled AROC for risky alcohol model was 0.4933 (95% CI = 0.492–0.498). The model of risky tobacco use returned an AROC of 0.8814 (95% CI = 0.8813–0.8815) and a F1 score of 0.67; the model accurately classified 54% of recent smokers and 93% of adolescents who had not smoked in the last month at a probability less than chance (*p* = 1.81 X **10**^**−46**^). Shuffled AROC for risky tobacco use was 0.495 (95% CI = 0.492–0.500). The AROC for lifetime cannabis use was of 0.9156 (95% CI = 0.9154–0.9157) and F1 score of 0.6039. Approximately 45% of adolescent lifetime cannabis users and 97% of non-users were correctly classified (*p* = 1.91 X **10**^**−9**^). The shuffled AROC for lifetime cannabis was 0.5005 (95% CI = 0.4911–0.5005). The model generated classifying risky cannabis users reported an AROC of 0.9247 (95% CI = 0.9245–0.9249) and F1 score of 0.4687; 31% of recent cannabis users and 99% of non-recent cannabis users were correctly classified at a level significantly better than chance (*p* = 2.90 X **10**^**−5**^). Shuffled AROC for risky cannabis model was 0.4976 (95% CI = 0.4907–0.5015). See Table 2 for Confusion Matrices.

**Table 2:** Confusion matrices.

Metrics	Model A: Lifetime Alcohol	Model B: Alcohol Risk	Model C: Tobacco Risk	Model D: Cannabis Lifetime	Model E: Cannabis Risk
True Positives	.8106	.6816	0.5437	0.4484	0.3100
False Positives	.1894	.3184	0.4563	0.5516	0.6900
True Negatives	.8129	.9079	0.9329	0.9670	0.9837
False Negatives	.1871	.0921	0.0671	0.0330	0.0127
Precision	.8106	.6816	0.5437	0.4484	0.3100
Recall	.8326	.8810	0.8979	0.9314	0.9606
AROC	.8924	.9051	0.8814	0.9156	0.9247
F1 Score	.8101	.7688	0.6723	0.6039	0.4687

### Domain contribution

**Fig 1:**
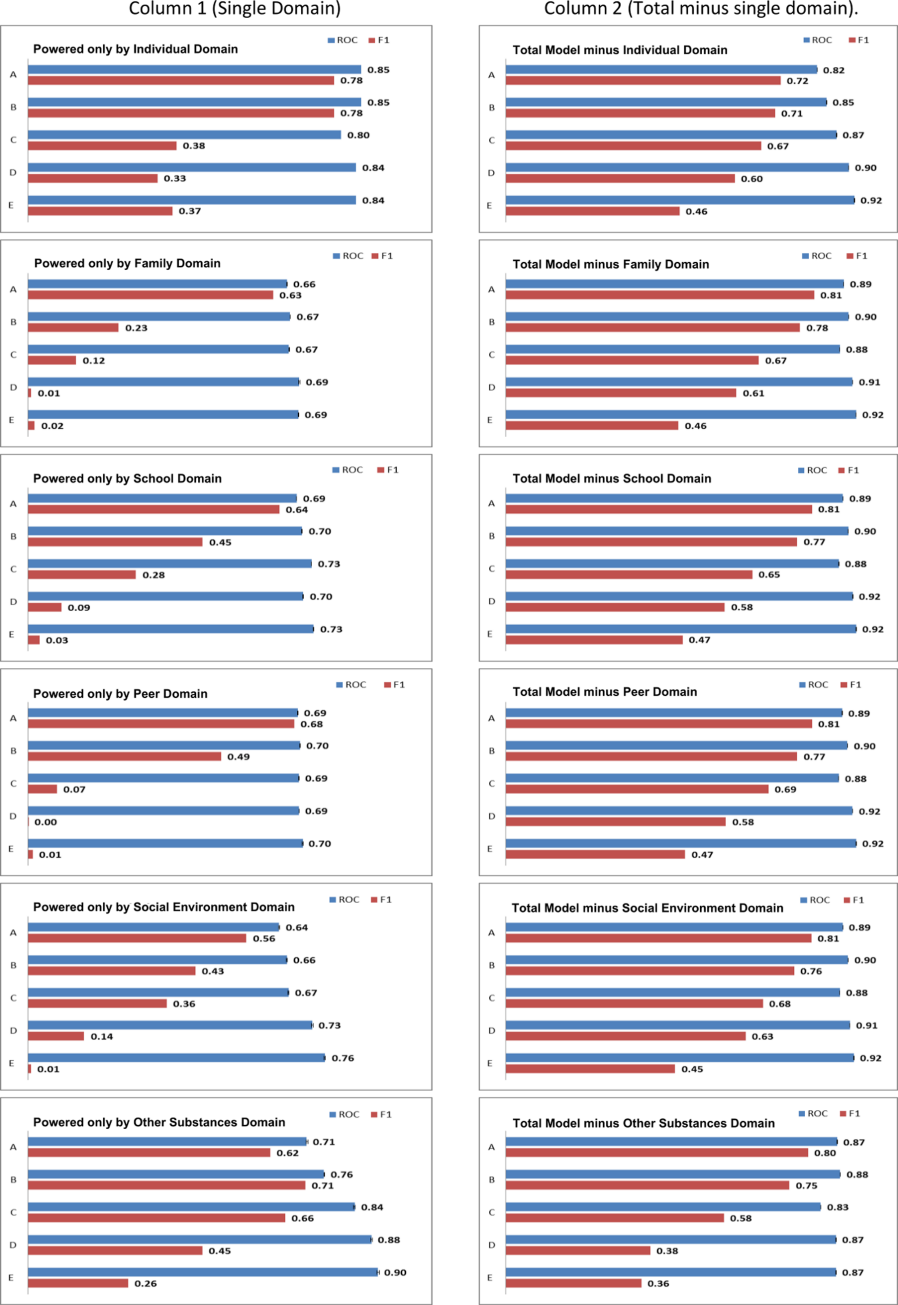
Model performance.

The performance of each model i) using only a single domain and ii) with individual domains removed is displayed in Fig 1. Domain analyses were included because they allow quantification of the importance that each domain of predictors had in the development of an overall predictive model. For example, readers may pay attention to how well a model performs when a domain of predictors are removed from the model and when only a domain of predictors are included in the model. For example, in Fig 1 under the column “Single Domain’, we can see that the family domain performs relatively poorly in predicting Model B when it is the only domain of predictors included. Conversely, under the column “Single Domain’, we can see that the individual domain alone performs well in predicting lifetime alcohol use (Model A). The ROC and F1 score in Fig 1 can also be compared with the overall performance of the total models presented in Table 2. Column 1 (powered by a single domain) reflects model performance when powered only by a single domain, separately showing how the Independent, Family, School, Peer, Social Environment and Polysubstance domains individually contribute to lifetime alcohol use (Model A); risky alcohol use (Model B); risky tobacco use (Model C); lifetime cannabis use (Model D) and risky cannabis use (Model E). Models A–E are visible on the y-axis of each figure in both Columns. Column 2 (total model minus single domain) quantifies the impact of overall model performance with a single domain removed.

Model performance when i) powered by a single domain and ii) where a different domain is iteratively removed is presented in Fig 1. Column 1 (powered by a single domain) captures model performance when powered only by the Independent, Family, School, Peer, Social Environment and Polysubstance domains. The x-axis reflects performance as measured by AROC and F1 score. The y-axis measures model performance for: lifetime alcohol use (A); risky alcohol use (B); risky tobacco use (C); lifetime cannabis use (D) and risky cannabis use (E).

For Model A, each domain classifies lifetime alcohol use at a level better than chance (AROC > 0.5) and the removal of any one domain does not substantially impair the model. However, the high AROC of the Individual domain (0.8524; 95% CI = 0.8522–0.8524) indicates that this domain contributes most to the model. In Model B, the AROC and F1 scores for model in which individual domains have been removed exhibit a narrow range of values (AROC: 0.85–0.90; F1: 0.71–0.78). However, individual domain performance exhibited substantial variation, with F1 scores of School, Peer and Social Environment ranging between 0.43–0.49 as well as the Family domain reporting 0.23. In Model C, removal of the Polysubstance domain reduced the F1 score by .09 –no other domain impacted model performance by greater/less than 0.02. The Polysubstance domain performed comparable to the main model (F1 = 0.66) with the School, Peer and Social Environment domains providing substantial predictive power with an F1 score of 0.28, 0.07 and 0.35 respectively. The Peer domain had limited predictive power with an F1 score of 0.07. The removal of the Polysubstance domain was the sole domain which substantially impaired Model D performance (F1 = .38). When models were constructed using individual domains alone, the Polysubstance domain reported the highest F1 score (0.45), followed by Individual (0.33) and Social Environment (0.14)–Peer, School and Family were substantially less (0.01–0.11). Model E performance was substantially impacted by the removal of the Polysubstance domain (F1 = 0.36). The Individual and Polysubstance domains were the only domains which provided individual predictive power with an F1 score of 0.37 and 0.26 respectively; all other domains reported an F1 score approaching zero.

#### Individual predictors

**Fig 2:**
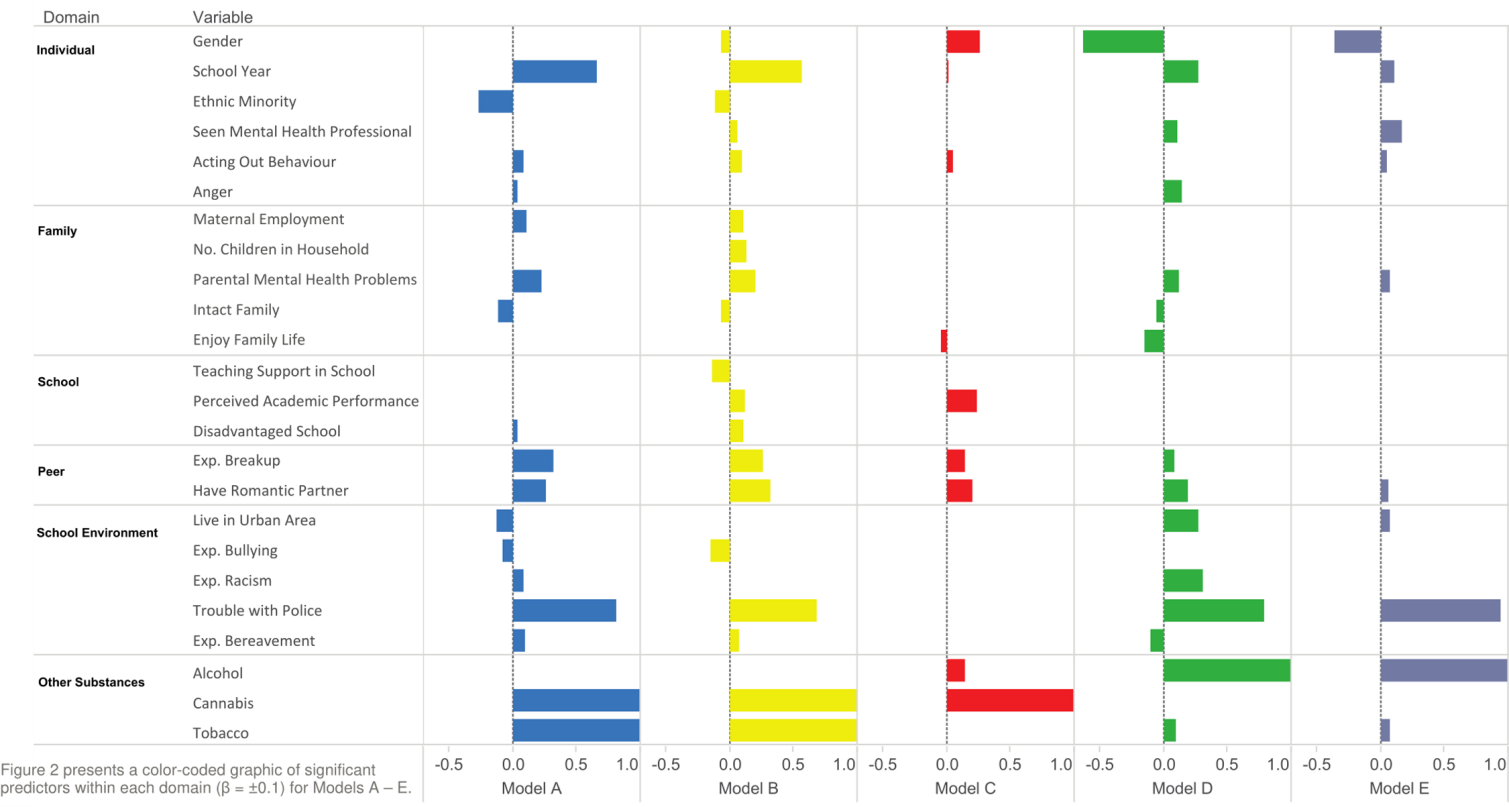
Beta coefficients by model.

The aim of the study was to identify the profile of adolescent substance misusers across multiple domains. Consequently, particular attention was paid to individual risk and protective factors which constituted the total models used to classify subjects. Fig 2 presents a color-coded graphic of predictors (β = ±0.1) across all outcomes; a complete list of beta values contributing to each model, and each model when a single domain is removed, can be found in S1–S5 Tables.

Fig 2 presents a color-coded graphic of predictors within each domain (β = ±0.1) for Models A–E. A differential substance misuse profile is visible in Fig 2. Gender (being female) beta coefficients have a strong negative association with cannabis models (D:β = -0.63; E:β = -0.35) and a positive association with the tobacco model (C:β = 0.26). School year is moderately predictive of alcohol use (A:β = 0.66; B:β = 0.57), weakly predictive of cannabis use (D:β = 0.27; E:β = 0.10) and unassociated with tobacco (C:β = 0.02). Ethnicity beta weights (identifying as non-white) are negatively related to alcohol use models only (A:β = -0.27; B:β = -0.12). Having accessed a MHP was a substantial predictor of both cannabis models (D:β = 0.11; E:β = 0.17), while anger had a beta coefficient only for lifetime cannabis use (β = 0.15). Acting out behavior was a predictor of risky alcohol use alone (B:β = 0.10). Maternal employment was positively associated with both alcohol models (A:β = 0.11; B:β = 0.11), being in a household with >3 children was associated with risky alcohol use (β = 0.13). Parental mental health problems were associated with both alcohol models and cannabis lifetime use (A:β = 0.22; B:β = 0.20; D:β = 0.12). Intact family was negatively associated with lifetime alcohol use (A:β = -0.12). Enjoyment of family life was negatively associated with lifetime cannabis use (D:β = -0.15).

Additional teaching support in school reduced the likelihood of scoring >5 on the AUDIT (B:β = -0.14), while being in a economically disadvantaged school increased the probability (B:β = 0.11). Lower academic performance was a predictive variable in the risky alcohol and risky tobacco model (B:β = 0.12; C:β = 0.23). Experience of a romantic breakup was positively associated with alcohol models and risky tobacco misuse (A:β = 0.32; B:β = 0.26; C:β = 0.14). Involvement with a romantic partner was predictive in all models bar risky cannabis (A:β = 0.26; B:β = 0.32; C:β = 0.20; D:β = 0.19). Living in an urban area decreased the likelihood of lifetime alcohol use (β = -0.13) and increased the likelihood of cannabis initiation (β = 0.27). Experience of racism was associated with a higher risk of having used cannabis (β = 0.31). Experience of bereavement increased the likelihood of having used alcohol (β = 0.10) but decreased the chance of having used cannabis (β = -0.11). Experience of trouble with the police was an important predictor of both alcohol and cannabis use (A: β = 0.81; B: β = 0.68; D: β = 0.79; E: β = 0.94). Finally, polysubstance use was a uniform predictor of substance use. For lifetime alcohol, both tobacco (β = 0.99) and cannabis use (β = 1.45) were predictors; a similar pattern was observed for risky alcohol use (β = 1.03; β = 1.21). For the cannabis lifetime model, alcohol use was a minor contributor (β = 0.10) while risky tobacco use was a predictor (β = 1.35). Both alcohol and cannabis use increased the likelihood of having smoked tobacco in the last month (β = 0.14 and β = 1.21, respectively). Likewise, risky tobacco use was a predictor of risky cannabis use (β = 1.75).

## Discussion

Using a machine learning approach, we identified common predictors of substance use as well as unique predictors, which differentially classified adolescent alcohol, tobacco and cannabis users–a pattern evident at the domain and individual predictor level. Each domain played a comparable role in predicting alcohol use, most domains played at least a substantial role in classifying tobacco users while cannabis use was predominantly influenced by the individual and polysubstance domains alone. At the level of individual predictors, polysubstance, externalizing behavior and peer relations were common features across all substances while differential patterns were observed for multiple variables across the individual, school, family and social domains, particularly for gender and school year. These results suggest that while common factors may underlie substance use, it is equally evident that individual predictors, across ecological domains, differentially characterize the use of different substances. Furthermore, given the extremely accurate and replicable models of substance use, which have been generated using a nationally, representative sample of Irish adolescents, we are confident in the replicability of these findings.

When observing domain contribution to model performance across substances, striking differences are observable. The importance of individual and polysubstance domains across substances contrasts with variation for family, school, peer and social domains. The stability of the individual and polysubstance domains across models may support previous studies, which have found a latent factor implicated in the initiation and persistent use of alcohol, tobacco and cannabis [23,24,28]. However, the equally noticeable variation in the role which individual predictors play in predicting specific substance use supports a growing consensus that environmental factors play a more substantial role than previously thought in shaping substance use; particularly during adolescence and especially for alcohol use [38, 39]. These findings support previous research, which has found that specific substances are associated with specific risk and protective factors [40]. Researchers have argued that because polysubstance use is high among adolescents, multi-substance prevention programs that focus on preventing substance use more globally should be implemented [41]. However, given the dissociable profiles of adolescent substance users, prevention strategies may be more effective if they try to understand the predictors unique to substance use.

This study’s unique research design, which allows interrogation of a high number of variables allows the detection of more complex patterns underpinning adolescent substance use. Results show that, in addition to common factors, unique factors across multiple ecological domains characterize ATC use; significantly, features (gender, school year, residence, experience of bereavement) differ in both the strength and direction of association across substances. A key limitation of the current research was that the My World Survey included assessments of lifetime alcohol and cannabis use, but not tobacco use, so the contribution of ecological domains towards models of lifetime tobacco use could not be determined in this study. These findings speak to both genetic-based research [24] and studies which deploy ecological frameworks [7,21] by identifying individual factors associated with substance use and emphasizing the importance of deploying a research design, which is capable of capturing the complex patterns underpinning adolescent substance use.

The importance of robust multivariable designs may be reflected in the failure of a number of well-documented findings to be replicated. Previous studies have found: psychological distress, such as depression, anxiety and stress [11]; dysfunctional coping strategies [13]; life satisfaction [42]; optimism [43] and self-esteem [12] to be associated with substance use in adolescence. However, these factors are effectively absent from each model of substance use in the current study, suggesting that their significance may be an artefact of the much smaller number of control variables used in previous studies. Once more, this suggests that research designs capable of modelling the large number of intercorrelated variables associated with substance use is critical to capturing complex patterns of substance use in adolescence.

However, these data also provide support for numerous ATC predictors highlighted by previous research. The current study found that clusters of high risk behaviors (such as acting-out behavior, poor perceived academic performance, trouble with police, socially-economically disadvantaged school) [44, 45] in addition to experiences of romantic relationships/breakups [5] were differentially associated substance use. These findings suggest that peer networks may be of particular importance in understanding substance use [46]. For alcohol use, important variables included: non-intact family [44]; living in a rural area [47]; experiencing bereavement [48]; and experiencing romantic relationships/breakups [5,18]. Tobacco use was linked to poor academic performance [49]. Finally, our research supports an association between cannabis use and male gender [50], living in an urban area, and anger [51].

Our study applied regularized logistic regression, via the Elastic Net, to a high dimension data-set of identified risk and protective factor based on a large nationally representative sample of adolescents. Multivariable predictive models reported out-of-sample performance using ten-fold cross-validation, rating model performance by efficiency in classifying new subjects. These significant strengths, however, must be considered alongside limitations. Cross-sectional data preclude the ability to make causal inferences. Further, statistical models reported in this study did account for variability attributable to the effect of school on substance use. The effect of school was omitted because such adjustment is not well-suited to out-of-sample validation. However, we believe that the demonstrable accuracy of the models reported in this study, which were cross-validated within a large national sample, increase confidence that such as omission is justified and do not reduce confidence in the results reported in this paper. Additionally, it would have been valuable to measure known correlates of substance use, such as impulsivity; association with deviant peer; parental substance use or religiosity, directly. Also, it should be noted that model performance was variable and readers should keep in mind when interpreting results that the model predicting risky cannabis use performed sub-optimally in successfully classifying adolescents who engaged in risky cannabis use; thus, risk and protective factors for this model are more relevant in identifying adolescents who do not engage in risky cannabis use. Finally, although single-item questions have ethical and practical advantages over more comprehensive measures [52], it is likely that single-item questions used in the current study (e.g., anger) are less reliable and valid than an established multi-question measure.

## Conclusions

Given the cross-sectional nature of the data, no light can be shed on causal relationships. However, an important conclusion can be drawn from the replicable and robust models of substance use presented in this study. The association between environmental factors and ATC use is more complex than previously thought. Although substantial commonalities were evident, different substances are also characterized by diverging sets of predictors, occasionally demonstrating differences in both strength and direction of association across substances. These findings support previous studies, which caution against assuming a uniform environmental effect on substance use and underline the importance of future studies employing research designs capable of capturing such differential patterns.

## Supporting information

S1 File Supplementary methodological information. (DOCX)

S2 File Alcohol Lifetime 1 Subanalysis.xlsx
Alcohol Risk A5 Subanalysis.xlsx
Cannabis Lifetime Subanalysis.xlsx
Cannabis Risk Subanalysis.xlsx
Nicotine Risk Subanalysis.xlsx
(ZIP)

S1 Table Alcohol lifetime. (DOCX)

S2 Table Alcohol risk. (DOCX)

S3 Table Tobacco risk. (DOCX)

S4 Table Cannabis lifetime. (DOCX)

S5 Table Cannabis risk. (DOCX)
