# Supplementary material for: Dissociable psychosocial profiles of adolescent substance users
Source: PLoS One. 2018 Aug 30;13(8):e0202498. doi: 10.1371/journal.pone.0202498 (PMC6116932; doi:10.1371/journal.pone.0202498)
Supplement: S1 File — (DOCX) [file pone.0202498.s001.docx]

# Supplementary Information

Full details of the procedures employed in the MWS-SL, including details on ethics, recruitment, data collection procedure and measures are available to view in the main methodological paper.^1^ Supplementary information presented below includes 1) any additional measures not included in the main method paper and 2) expanded description of the machine learning analytical procedure.

## Measures in the Individual Domain

Single items were used to assess participants’ gender, school year, ethnicity, whether participants’ have seen a mental health professional, feelings of anger, and body satisfaction.

Ethnic minority. Ethnicity was captured using the item “In terms of your ethnic or cultural background are you:” White, Black, Asian, Irish Traveller or Other. Participants were scored according to their ethnic membership as (0) those who identified themselves as White, or (1) those who identified themselves as Black, Asian, Irish Traveller or Other.

Seen mental health professional. Participants were asked “have you ever seen a mental health professional?” Response alternatives were (0) no, or (1) yes.

Anger. Feeling angry was measured with the item “do you feel angry a lot?” with response alternatives of (i) no, (ii) sometimes, or (iii) yes, scored respectively as 0, 1 and 2.

Body dissatisfaction. This was measured with the item “how satisfied are you with your body?” Response alternatives were (i) very dissatisfied, (ii) dissatisfied, (iii) neither satisfied or dissatisfied, (iv) satisfied, or (v) very satisfied. These five categories were merged to form three categories: (0) dissatisfied (1) neither satisfied or dissatisfied, or (2) satisfied.

**Experiences of psychotic symptoms**. A shortened three-item version of the Adolescent Psychotic-like Symptom Screener (APSS)^2^ assessed a variety of psychotic symptoms in a brief period of time. Three questions on visual hallucinations, auditory hallucinations and delusions from the APSS, with the greatest predictive power of psychotic symptoms, were included. Participants selected one of three responses: no (scored 0), maybe (scored 0.5) or yes, definitely (scored 1). Higher scores were indicative of greater experiences of psychotic symptoms and total scores ranged from 0 to 3. Cronbach’s alpha for the APSS in the present study was .75.

Social competence. The Social Competence five-item subscale of the Resilience Scale for Adolescence (READ)^3^ assessed social adeptness, cheerfulness, communication skills and flexibility in social situations. Participants were asked to indicate the degree to which they agree with statements such as ‘I am good at talking to new people’ and ‘I easily make others feel comfortable around me’. Response alternatives included: (i) totally disagree, (ii) disagree, (iii) neither agree or disagree, or (iv) agree, or (v) totally agree. Total scores ranged from 5 to 25. Cronbach’s alpha for the READ-Social Competence Subscale in the present study was .74.

Acting out behaviour. A seven-item version of the Behavioral Adjustment Scale (BAS)^4^ assessed the frequency over the past month that participants engaged in deviant acting out behaviour such as cutting a class or skipping school, had been suspended or expelled from school, or cheated on an exam. Response alternatives included: (i) never, (ii) once or twice, (iii) 3 or 4 times, (iv) pretty often, or (v) almost every day. Cronbach’s alpha for this measure was .77. Total scores ranged from 7 to 35.

## Measures in the Family Domain

The family domain included single items such as maternal employment, paternal employment, maternal education, paternal education, living in an intact family, number of children in household, parental mental health problems, and enjoyment of family life.

Maternal employment. Participants were asked “is your mother:” (i) Employed full-time/part-time, (ii) Unemployed, or (iii) Stay-at-home Mother. For the variable ‘Mother Employed Versus Other’, participants were grouped according to whether their mother was unemployed or stayed-at-home (0) or whether their mother was employed (1). For the variable ‘Stay-at-home Mother Versus Other’, participants were grouped according to whether their mother was unemployed or employed (0) or whether their mother stayed-at-home (1).

Paternal employment. Participants were asked “is your father:” (1) Employed full-time/part-time, (2) Unemployed, or (3) Stay-at-home Dad. These employment groups were merged to form two categories of father unemployed or stay-at-home Dad (0) or father employed full-time/part-time (1) and was labelled as 'Father Employed Versus Other'.

**Maternal education**. This was measured with the item “what is the highest educational level of your mother?” Response alternatives were (i) Junior Certificate, (ii) Leaving Certificate, (iii) Qualified Tradesperson, (iv) College/ University Degree (v) Professional Degree or (vi) Don’t Know. These educational/qualification groups were merged to form two larger categories of declared lower second-level education (0) (i.e., Junior Certificate), or completed, at least, second level education (1).

**Father’s highest level of education**. This item was similar to maternal education and a binary variable similar to maternal education was created.

Intact family. This was measured by asking participants to respond to the statement “My parents are:” (i) Married, (ii) Separated, (iii) Single, (iv) Divorced, (v) Living together but not married, (vi) Remarried, (vii) Deceased, or (viii) Other. These groups were merged to form two larger categories of intact family defined by ‘married’ and ‘living together but not married’ (1), or non-intact family defined by all other response alternatives (0).

No. children in household. The number of child in each household was measured by asking participants to respond to the statement “Number of children in your family including yourself" with options of ‘1-3’, ‘4-5’, or ‘6 or more’. Each answer reflects a different level of the independent variable was scored as 0, 1 and 2, respectively.

**Parental mental health problems** were measured by asking “Has your mother or father ever had a mental health problem?” Response alternatives included (i) no, (ii) don’t know or (iii) yes. These three categories were merged to form two categories of no/don’t know which was scored as 0, or (ii) yes which was scored as 1.

**Family cohesion**. The READ Family Cohesion six-item subscale assessed support within the family and the family’s ability to maintain a positive outlook.^3^ Participants were asked to indicate the degree to which they agree with statements such as ‘In my family we share views of what is important’ and ‘My family view the future as positive even when very sad things happen’. Response alternatives were on a 5-point likert scale ranging from (i) totally disagree to (v) totally agree. Total scores ranged from 6 to 30. Cronbach’s alpha for the READ-Family Cohesion Subscale in the present study was .86.

**Enjoy family life** was measured with the question “Do you enjoy your family life?” Response alternatives included (i) no, (ii) sometimes, or (iii) yes. Each answer reflects a different level of the independent variable was scored as 0, 1 and 2, respectively.

## Measures in the School Domain

**Teaching Support in school** was measured by asking “do you receive any additional teaching support at school?” Response alternatives were no (0), or yes (1).

**Perceived academic position** was measured with the statement: “In my school work I am:”, to which participants could respond, (i) at the top of the class, (ii) middle of the class, or (iii) at the bottom. Each answer reflects a different level of the independent variable was scored as 0, 1 and 2, respectively.

**Disadvantaged school**. Based on the Department of Education’s published list of schools were classified as economically disadvantaged (0) or non-disadvantaged (1).

**Mixed school.** Based on the Department of Education’s published list of schools, schools were classified as (i) single-sex girls, (ii) single-sex boys or (iii) mixed gender. Single-sex schools were coded as 0 and mixed schools were coded as 1.

**School connectedness**. The Hemingway MAC-School Connectedness [Karcher, unpublished data] subscale contains six statements and measures the importance participant’s place in school (i.e., how much they care for school) and the degree to which participants become actively involved in being successful in school. Response alternatives were (i) not at all, (ii) not really, (iii) sort of, (iv) true, or (v) very true. Values ranged from 6 to 30. Cronbach’s alpha for the Hemingway MAC-School Connectedness in the present study was .82.

**Teacher connectedness.** The Hemingway, MAC-Teacher Connectedness [Karcher, unpublished data] subscale contains five statements and assesses participants’ degree of concern about their relationships with their teachers, their sense of enjoying being with teachers and their degree of affective involvement in their relationships with their teachers. Values ranged from 6 to 30. Alpha for the Hemingway MAC-Teacher Connectedness in the present study was .83.

## Measures in the Peer Domain

**Romantic relationship breakup** was measured by asking “Have you ever experienced a breakup?” Response alternatives were (i) never, (ii) yes, it happened more than a year ago, or (iii) yes, it happened within the past year which were scored as 0, 1 and 2 respectively.

**Involvement with romantic partner** was measured with the question “do you currently have a boyfriend/girlfriend or romantic partner?” Response alternatives were no (0), or yes (1).

**Perceived peer support** was measured using the Multidimensional Scale of Perceived Social Support (MSPSS) ‘Friend’ subscale.^5^ Adolescents responded to four questions in which they evaluated their agreement with statements, such as ‘My friends really try to help me’, using a seven point likert scale ranging from ‘very strongly agree’ to ‘very strongly disagree’. Scores ranged from 4 to 28. Cronbach’s alpha for the MSPSS ‘Friend’ subscale in present study was .93.

**Peer connectedness**. The Hemingway, Measure of Adolescent Connectedness (MAC)-Peer Connectedness [Karcher, unpublished data] subscale contains five statements that ask participants to rate degree to which he/she enjoys working with peers on school-related tasks and projects. The subscale assessed the degree to which participants feel they fit in with their peers, their sense of belonging in the school in general, and feelings of acceptance. Response alternatives were (i) not at all, (ii) not really, (iii) sort of, (iv) true, or (v) very true. Values ranged from 6 to 30. Cronbach’s alpha for the Hemingway MAC-Peer Connectedness in the present study was .71.

## Measures in the Socio Environment Domain

**Safe neighbourhood.** Participants were asked to respond to the question “How safe do you feel living in your neighbourhood?” on a seven-point likert scale ranging from (i) very unsafe to (vii) very safe.

**Live in Urban Area.** Residence was measured by asking “Where are you living?” Response alternatives included: (i) in a city, (ii) in a town, (iii) in a village, (iv) in the countryside outside a town/city, (v) on a farm, or (vi) other. These six categories were merged to form two larger categories of those who live in a village, countryside, outside a town, on a farm or other (0) or ‘urban’ for those who live in a city/in a town (1).

**Experienced racism** was measured with the item “Have you ever been treated unfairly because of your race or ethnic group?” Response alternatives were no (0), or yes (1).

**Experienced bullying**. “Have you ever been bullied?” Response alternatives were no (0), or yes (1).

**Trouble with police**. “Have you ever been in trouble with the police?” Response alternatives were no (0), or yes (1).

**Informal help seeking**. Informal help-seeking was assessed with 6 items adapted from Saunders et al.^6^ Participants were asked who they would talk to first if they had problems with (i) family, (ii) a friend, (iii) a boyfriend or girlfriend, (iv) school, (v) depression, or (vi) alcohol and drug use. Each of the 6 items were coded into a binary variable where a score of 0 was assigned to ‘no-one’ , while a score of 1 was assigned to responses related to seeking help from family, friend, or other; yielding score ranges of 0 to 6.

**One good adult**. Participants were asked to indicate whether or not they agreed with the statement “There is a special adult who is around when I am in need” on a seven-point likert scale ranging (i) very strongly disagree to (vii) very strongly agree.

**Experienced bereavement**. “Has anyone close to you died in the last 12 months?” Response alternatives were no (0), or yes (1).

**Machine Learning Analytical Procedure**

### We conducted logistic regression with elastic net regularization. Elastic Net regularization imposes a hybrid of both L_1_- and L_2_-norm penalties (i.e., penalties on the absolute (L_1_ norm) and squared values of the β weights (L_2_ norm)).^7^ This allows relevant but correlated coefficients to coexist in a sparse model fit, by doing automatic variable selection and continuous shrinkage simultaneously, and selects or rejects groups of correlated variables. Least absolute shrinkage and selection operator (LASSO)^8^ and ridge regression^9^ are special cases of the Elastic Net. All predictor data were first feature scaled (z-score transformed). We implemented ten-fold cross-validation with nested cross-validation for tuning and validating the model. Briefly, to implement cross-validation, the data were randomly split into 10 groups. A model was then generated based on 9 training groups, and then applied to the remaining independent testing group. Each group served as the testing group once, resulting in 10 different models, and predictions for every subject based on independent data. Nested cross-validation involved subdividing the 9 training groups (i.e., 90% of the sample) into a further 10 groups (‘inner’ folds). Within these 10 inner folds, 9 were utilized for training a model over a range of 30 alpha (.01-1) and 30 lambda (.0001-1) values. This generated a resulting model fit on the inner fold test set for each possible combination of alpha and lambda. The mean fit over all 10 inner folds for each combination of alpha and lambda was then calculated and then used to determine the optimal parameters for the outer fold. Using an AROC score, model performance can be categorized as: excellent (>0.9), very good (0.8–0.9), good (0.7–0.8), average (0.6-0.7) or poor (<0.6).^10^

**References**

1. Dooley B, Fitzgerald A. Methodology on the My World Survey (MWS): a unique window into the world of adolescents in Ireland. Early Interv Psychiatry. 2013;7(1):12-22.
2. Kelleher I, Harley M, Murtagh A, Cannon M. Are screening instruments valid for psychotic-like experiences? A validation study of screening questions for psychotic-like experiences using in-depth clinical interviews. Schizophr Bull. 2012;37(2):362-369.
3. Hjemdal O, Friborg O, Stiles TC, Martinussen M, Rosenvinge JH. A new rating scale for adolescent resilience: grasping the central protective resources behind healthy development. Meas Eval Couns Dev. 2006;39:84-96.
4. Brown BB, Clasen DR, Eicher SA. Perceptions of peer pressure, peer conformity dispositions, and self-reported behaviour among adolescents. Dev Psychol. 1986;22:521-530.
5. Zimet GD, Powell SS, Farley GK, Werkman S, Berkoff KA. Psychometric characteristics of the Multidimensional Scale of Perceived Social Support. J Pers Assess. 1990;55:610-617.
6. Saunders SM, Resnick MD, Hoberman HM, Blum RW. Formal help-seeking behaviour of adolescents identifying them- selves as having mental health problems. J Am Acad Child Adolesc Psychiatry. 1994;33:718-728.
7. Zou H Hastie T. Regularization and variable selection via the elastic net. J R Stat Soc Series B Stat Methodol. 2005;67(2):301-320.
8. Tibshirani R. Regression shrinkage and selection via the lasso. J R Stat Soc Series B Stat Methodol. 1996;58(1):267-288.
9. Hoerl AE, Kennard RW. Ridge regression: Biased estimation for nonorthogonal problems. Technometrics. 1970;12(1):55-67.
10. Metz CE. Basic principles of ROC analysis. Semin Nucl Med. 1978;8(4):283-298.
