# Supplementary material for: Dissociable psychosocial profiles of adolescent substance users
Source: PLoS One. 2018 Aug 30;13(8):e0202498. doi: 10.1371/journal.pone.0202498 (PMC6116932; doi:10.1371/journal.pone.0202498)
Supplement: S1 Table — (DOCX) [file pone.0202498.s003.docx]

| **Domain** | **Variable** | **Total** | **Individual** | **Family** | **School** | **Peer** | **Social Environment** | **Other Substances** | **Single Domain** |
| --- | --- | --- | --- | --- | --- | --- | --- | --- | --- |
| **Individual** | Gender |  |  |  | -0.06 |  |  |  | -0.24 |
|  | School Year | 0.66 |  | 0.66 | 0.64 | 0.69 | 0.63 | 0.70 | 0.77 |
|  | Ethnic Minority | -0.27 |  | -0.31 | -0.37 | -0.29 | -0.15 | -0.30 | -0.28 |
|  | Seen Mental Health Professional |  |  | 0.06 |  |  |  |  | 0.20 |
|  | Psychotic Symptoms |  |  |  |  |  |  |  |  |
|  | Depression |  |  |  |  |  |  |  |  |
|  | Anxiety |  |  |  | 0.00 |  |  |  |  |
|  | Stress |  |  |  |  |  |  |  | -0.01 |
|  | Avoidance Coping |  |  | 0.00 | 0.00 |  |  | 0.00 | 0.01 |
|  | Planning Coping | 0.00 |  | -0.01 | -0.01 | -0.01 |  | 0.00 | -0.03 |
|  | Support Coping | 0.02 |  | 0.02 | 0.02 | 0.03 | 0.02 | 0.03 | 0.05 |
|  | Anger | 0.03 |  | 0.06 | 0.05 | 0.05 | 0.03 | 0.06 | 0.17 |
|  | Body Dissatisfaction | -0.02 |  | -0.03 | -0.04 | -0.01 |  | -0.05 |  |
|  | Acting Out Behaviour | 0.08 |  | 0.08 | 0.10 | 0.09 | 0.09 | 0.10 | 0.20 |
|  | Satisfaction with Life |  |  | 0.00 | -0.01 |  |  |  | -0.04 |
|  | Optimism |  |  |  | 0.00 |  |  |  | -0.01 |
|  | READ – Social Competence | 0.02 |  | 0.02 | 0.02 | 0.04 | 0.03 | 0.03 | 0.06 |
|  | Self-esteem |  |  |  | 0.00 |  |  |  | -0.01 |
| **Family** | Maternal Employment | 0.11 | 0.09 |  | 0.12 | 0.12 | 0.10 | 0.10 |  |
|  | Stay-at-home Mother |  |  |  |  |  |  |  | -0.14 |
|  | Paternal Employment |  |  |  |  |  |  |  |  |
|  | Maternal Education |  | 0.02 |  |  |  |  | 0.01 | 0.02 |
|  | Paternal Education | 0.03 | 0.05 |  | 0.03 | 0.03 | 0.02 | 0.02 |  |
|  | No. Children in household |  |  |  | 0.04 |  |  |  | 0.06 |
|  | Parental Mental Health Problems | 0.22 | 0.46 |  | 0.22 | 0.23 | 0.21 | 0.28 | 0.55 |
|  | Intact Family | -0.12 | -0.06 |  | -0.14 | -0.16 | -0.11 | -0.15 | -0.23 |
|  | Perceived family support | -0.01 | -0.02 |  | -0.01 |  | -0.01 | 0.00 | -0.02 |
|  | READ – Family Cohesion | -0.02 | -0.03 |  | -0.03 | -0.03 | -0.02 | -0.03 | -0.06 |
|  | Enjoy family life |  |  |  |  |  |  | -0.06 | -0.22 |
| **School** | Teaching Support in School |  | -0.12 | -0.09 |  | -0.07 |  | -0.08 | -0.13 |
|  | Perceived Academic Position |  |  | 0.03 |  | 0.04 |  | 0.09 | 0.24 |
|  | Disadvantaged School | 0.04 | 0.18 | 0.07 |  |  |  | 0.10 | 0.24 |
|  | Mixed School |  |  |  |  |  |  |  |  |
|  | School Connectedness | -0.03 | -0.04 | -0.03 |  | -0.03 | -0.03 | -0.04 | -0.08 |
|  | Teacher Connectedness | -0.04 | -0.03 | -0.05 |  | -0.04 | -0.05 | -0.05 | -0.06 |
| **Peer** | Exp. Breakup | 0.32 | 0.31 | 0.33 | 0.33 |  | 0.31 | 0.36 | 0.43 |
|  | Have Romantic Partner | 0.26 | 0.50 | 0.27 | 0.28 |  | 0.28 | 0.38 | 0.73 |
|  | Perceived Peer Support | 0.01 | 0.02 | 0.01 | 0.01 |  | 0.00 | 0.01 |  |
|  | Peer Connectedness |  |  |  | -0.01 |  |  |  | -0.04 |
| **Social Environment** | Safe Neighbourhood |  |  |  |  |  |  |  | -0.11 |
|  | Live in Urban area | -0.13 | -0.05 | -0.13 | -0.16 | -0.12 |  | -0.12 |  |
|  | Exp Racism | 0.08 | -0.06 | 0.11 | 0.13 | 0.13 |  | 0.15 | 0.06 |
|  | Exp Bullying | -0.08 | -0.08 | -0.09 | -0.17 | -0.05 |  | -0.09 |  |
|  | Trouble with Police | 0.81 | 0.81 | 0.84 | 0.88 | 0.86 |  | 1.06 | 1.18 |
|  | Inform |  |  |  |  |  |  |  | -0.05 |

| **Domain** | **Variable** | **Total** | **Individual** | **Family** | **School** | **Peer** | **Social Environment** | **Other Substances** | **Single Domain** |
| --- | --- | --- | --- | --- | --- | --- | --- | --- | --- |
| **Social Environment** | One Good Adult | -0.02 |  | -0.05 | -0.03 | -0.03 |  | -0.05 | -0.11 |
|  | Exp bereavement | 0.10 |  | 0.10 | 0.12 | 0.13 |  | 0.11 | 0.08 |
| **Other Substances** | Tobacco | 0.99 | 1.63 | 1.03 | 1.00 | 1.07 | 1.05 |  | 0.19 |
|  | Cannabis | 1.45 | 1.77 | 1.46 | 1.46 | 1.58 | 1.44 |  | 0.27 |
| **Model Performance** | AROC | 0.89 | 0.82 | 0.89 | 0.89 | 0.89 | 0.89 | 0.87 |  |
|  | Lower | 0.89 | 0.82 | 0.89 | 0.89 | 0.89 | 0.89 | 0.87 |  |
|  | Upper | 0.89 | 0.82 | 0.89 | 0.89 | 0.89 | 0.89 | 0.88 |  |
|  | F1 Score | 0.81 | 0.72 | 0.81 | 0.81 | 0.81 | 0.81 | 0.80 |  |
