# Supplementary material for: Dissociable psychosocial profiles of adolescent substance users
Source: PLoS One. 2018 Aug 30;13(8):e0202498. doi: 10.1371/journal.pone.0202498 (PMC6116932; doi:10.1371/journal.pone.0202498)
Supplement: S2 Table — (DOCX) [file pone.0202498.s004.docx]

| **Domain** | **Variable** | **Total** | **Individual** | **Family** | **School** | **Peer** | **Social Environment** | **Other Substances** | **Single Domain** |
| --- | --- | --- | --- | --- | --- | --- | --- | --- | --- |
| **Individual** | Gender | -0.06 |  |  | -0.08 | -0.07 | -0.12 | -0.05 | -0.24 |
|  | School Year | 0.57 |  | 0.57 | 0.58 | 0.56 | 0.57 | 0.68 | 0.77 |
|  | Ethnic Minority | -0.12 |  | -0.11 | -0.16 | -0.10 |  | -0.13 | -0.28 |
|  | Seen Mental Health Professional | 0.07 |  | 0.10 | 0.06 | 0.08 | 0.08 | 0.13 | 0.20 |
|  | Psychotic Symptoms |  |  |  |  |  |  |  |  |
|  | Depression | 0.00 |  | 0.00 | 0.00 | 0.00 | 0.00 | 0.01 |  |
|  | Anxiety |  |  |  |  |  |  |  |  |
|  | Stress | 0.00 |  | 0.00 |  | 0.00 | 0.00 |  | -0.01 |
|  | Avoidance Coping | 0.01 |  | 0.01 | 0.01 | 0.01 | 0.01 | 0.01 | 0.01 |
|  | Planning Coping |  |  |  | -0.01 |  |  |  | -0.03 |
|  | Support Coping | 0.02 |  | 0.02 | 0.02 | 0.03 | 0.02 | 0.03 | 0.05 |
|  | Anger |  |  |  |  |  |  | 0.03 | 0.17 |
|  | Body Dissatisfaction |  |  |  | -0.01 |  |  | -0.02 | -0.02 |
|  | Acting Out Behaviour | 0.10 |  | 0.10 | 0.12 | 0.10 | 0.11 | 0.13 | 0.20 |
|  | Satisfaction with Life | 0.00 |  | -0.01 | -0.01 | 0.00 | 0.00 | -0.01 | -0.04 |
|  | Optimism |  |  |  |  |  |  |  | -0.01 |
|  | READ – Social Competence | 0.02 |  | 0.02 | 0.02 | 0.03 | 0.03 | 0.03 | 0.06 |
|  | Self-esteem |  |  |  |  |  |  |  | -0.01 |
| **Family** | Maternal Employment | 0.11 | 0.10 |  | 0.12 | 0.11 | 0.12 | 0.11 |  |
|  | Stay-at-home Mother |  |  |  |  |  |  |  | -0.14 |
|  | Paternal Employment |  |  |  |  |  |  |  | -0.06 |
|  | Maternal Education |  | 0.03 |  |  |  |  | 0.02 |  |
|  | Paternal Education |  | 0.01 |  |  |  |  |  |  |
|  | No. Children in household | 0.13 | 0.11 |  | 0.13 | 0.12 | 0.14 | 0.12 | 0.13 |
|  | Parental Mental Health Problems | 0.20 | 0.36 |  | 0.19 | 0.23 | 0.20 | 0.24 | 0.51 |
|  | Intact Family | -0.07 | -0.04 |  | -0.07 | -0.09 | -0.09 | -0.12 | -0.22 |
|  | Perceived family support | -0.01 | -0.02 |  | -0.01 | -0.01 | -0.01 | 0.00 | -0.03 |
|  | READ – Family Cohesion | -0.01 | -0.02 |  | -0.02 | -0.01 | -0.01 | -0.02 | -0.05 |
|  | Enjoy Family Life |  |  |  |  |  |  |  | -0.21 |
| **School** | Teaching Support in School | -0.14 | -0.17 | -0.13 |  | -0.15 | -0.10 | -0.16 | -0.14 |
|  | Perceived Academic Position | 0.12 | 0.14 | 0.12 |  | 0.15 | 0.14 | 0.19 | 0.37 |
|  | Disadvantaged School | 0.11 | 0.22 | 0.10 |  | 0.10 | 0.09 | 0.18 | 0.28 |
|  | Mixed School | -0.08 | -0.15 | -0.07 |  | -0.07 | -0.10 | -0.08 | -0.15 |
|  | School Connectedness | -0.02 | -0.04 | -0.02 |  | -0.03 | -0.02 | -0.04 | -0.08 |
|  | Teacher Connectedness | -0.03 | -0.03 | -0.03 |  | -0.03 | -0.03 | -0.03 | -0.06 |
| **Peer** | Exp. Breakup | 0.26 | 0.26 | 0.26 | 0.28 |  | 0.26 | 0.34 | 0.39 |
|  | Have Romantic Partner | 0.32 | 0.47 | 0.33 | 0.33 |  | 0.34 | 0.44 | 0.72 |
|  | Perceived Peer Support |  | 0.01 |  |  |  |  |  |  |
|  | Peer Connectedness |  |  |  |  |  |  |  | -0.03 |
| **Social Environment** | Safe Neighbourhood | 0.03 |  | 0.03 | 0.03 | 0.02 |  | 0.06 | -0.06 |
|  | Live in Urban area |  |  |  |  |  |  |  |  |
|  | Exp Racism |  | -0.13 |  |  |  |  |  |  |
|  | Exp Bullying | -0.15 | -0.10 |  | -0.20 | -0.12 |  | -0.16 |  |
|  | Trouble with Police | 0.68 | 0.70 | 0.69 | 0.72 | 0.69 |  | 1.03 | 1.30 |
|  | Inform |  |  |  |  |  |  |  | -0.05 |

| **Domain** | **Variable** | **Total** | **Individual** | **Family** | **School** | **Peer** | **Social Environment** | **Other Substances** | **Single Domain** |
| --- | --- | --- | --- | --- | --- | --- | --- | --- | --- |
| **Social Environment** | One Good Adult | -0.04 | -0.03 | -0.06 | -0.04 | -0.04 |  | -0.05 | -0.12 |
|  | Exp bereavement | 0.08 |  | 0.08 | 0.09 | 0.11 |  | 0.10 | 0.08 |
| **Other Substances** | Tobacco | 1.03 | 1.42 | 1.04 | 1.05 | 1.07 | 1.15 |  | 0.42 |
|  | Cannabis | 1.21 | 1.39 | 1.21 | 1.27 | 1.25 | 1.25 |  | 0.40 |
| **Model Performance** | AROC | 0.91 | 0.85 | 0.90 | 0.90 | 0.90 | 0.90 | 0.88 |  |
|  | Lower | 0.91 | 0.85 | 0.90 | 0.90 | 0.90 | 0.90 | 0.88 |  |
|  | Upper | 0.91 | 0.85 | 0.90 | 0.90 | 0.90 | 0.90 | 0.88 |  |
|  | F1 Score | 0.77 | 0.71 | 0.78 | 0.77 | 0.77 | 0.76 | 0.75 |  |
