# Supplementary material for: Dissociable psychosocial profiles of adolescent substance users
Source: PLoS One. 2018 Aug 30;13(8):e0202498. doi: 10.1371/journal.pone.0202498 (PMC6116932; doi:10.1371/journal.pone.0202498)
Supplement: S3 Table — (DOCX) [file pone.0202498.s005.docx]

| **Domain** | **Variable** | **Total** | **Individual** | **Family** | **School** | **Peer** | **Social Environment** | **Other Substances** | **Single Domain** |
| --- | --- | --- | --- | --- | --- | --- | --- | --- | --- |
| **Individual** | Gender | 0.26 |  | 0.26 | 0.19 | 0.30 | 0.27 | 0.25 | 0.12 |
|  | School Year | 0.02 |  |  |  | 0.03 | 0.01 | 0.27 | 0.33 |
|  | Ethnic Minority |  |  |  |  |  |  |  |  |
|  | Seen Mental Health Professional |  |  |  |  |  |  | 0.15 | 0.28 |
|  | Psychotic Symptoms |  |  |  |  |  |  |  |  |
|  | Depression |  |  |  |  |  |  |  |  |
|  | Anxiety |  |  |  |  |  |  |  |  |
|  | Stress |  |  |  |  |  |  |  |  |
|  | Avoidance Coping |  |  |  |  |  |  |  |  |
|  | Planning Coping |  |  |  |  |  |  |  | -0.02 |
|  | Support Coping |  |  |  |  |  |  | 0.02 | 0.03 |
|  | Anger |  |  |  |  |  |  | 0.08 | 0.17 |
|  | Body Dissatisfaction | -0.03 |  |  | -0.02 | -0.03 | -0.03 | -0.07 | -0.04 |
|  | Acting Out Behaviour | 0.05 |  | 0.05 | 0.06 | 0.05 | 0.05 | 0.10 | 0.16 |
|  | Satisfaction with Life |  |  |  |  |  |  | -0.01 | -0.03 |
|  | Optimism |  |  |  |  |  |  |  | -0.01 |
|  | READ – Social Competence |  |  |  |  |  |  | 0.03 | 0.05 |
|  | Self-esteem |  |  |  | -0.01 |  |  | -0.01 | -0.02 |
| **Family** | Maternal Employment |  |  |  |  |  |  |  |  |
|  | Stay-at-home Mother |  |  |  |  |  |  |  | -0.13 |
|  | Paternal Employment |  |  |  |  |  |  |  | -0.07 |
|  | Maternal Education |  |  |  |  |  |  | 0.01 |  |
|  | Paternal Education |  |  |  |  |  |  |  | -0.04 |
|  | No. Children in household |  |  |  |  |  |  |  | 0.06 |
|  | Parental Mental Health Problems |  |  |  |  |  |  |  | 0.29 |
|  | Intact Family |  |  |  |  |  |  | -0.09 | -0.22 |
|  | Perceived family support |  |  |  |  |  |  | 0.00 | -0.02 |
|  | READ – Family Competence |  |  |  |  |  |  | -0.01 | -0.04 |
|  | Enjoy Family Life | -0.04 | -0.08 |  |  | -0.07 | -0.04 | -0.13 | -0.32 |
| **School** | Teaching Support in School |  |  |  |  |  |  |  |  |
|  | Perceived Academic Position | 0.23 | 0.21 | 0.23 |  | 0.27 | 0.24 | 0.53 | 0.54 |
|  | Disadvantaged School |  |  |  |  |  |  | 0.24 | 0.25 |
|  | Mixed School |  |  |  |  |  |  |  |  |
|  | School Connectedness | -0.03 | -0.04 | -0.03 |  | -0.04 | -0.03 | -0.09 | -0.09 |
|  | Teacher Connectedness |  | -0.01 |  |  |  |  | -0.05 | -0.05 |
| **Peer** | Exp. Breakup | 0.14 | 0.12 | 0.14 | 0.14 |  | 0.14 | 0.26 | 0.37 |
|  | Have Romantic Partner | 0.20 | 0.21 | 0.20 | 0.20 |  | 0.21 | 0.41 | 0.68 |
|  | Perceived Peer Support |  |  |  |  |  |  | 0.01 |  |
|  | Peer Connectedness |  |  |  |  |  |  | 0.01 | -0.04 |
| **Social Environment** | Safe Neighbourhood |  |  |  |  |  |  |  | -0.07 |
|  | Live in Urban area |  |  |  |  |  |  |  | 0.02 |
|  | Exp Racism |  |  |  |  |  |  |  | 0.13 |
|  | Exp Bullying |  |  |  |  |  |  |  | 0.08 |
|  | Trouble with Police |  | 0.08 |  |  |  |  | 0.72 | 0.74 |
|  | Inform |  |  |  |  |  |  |  | -0.05 |

| **Domain** | **Variable** | **Total** | **Individual** | **Family** | **School** | **Peer** | **Social Environment** | **Other Substances** | **Single Domain** |
| --- | --- | --- | --- | --- | --- | --- | --- | --- | --- |
| **Social Environment** | One Good Adult |  |  |  |  |  |  | -0.04 | -0.07 |
|  | Exp Bereavement |  |  |  |  |  |  | 0.04 | 0.11 |
| **Other Substances** | Alcohol | 0.14 | 0.12 | 0.14 | 0.15 | 0.15 | 0.14 |  | 0.06 |
|  | Cannabis | 1.21 | 1.05 | 1.21 | 1.18 | 1.26 | 1.21 |  | 0.71 |
| **Model Performance** | AROC | 0.88 | 0.87 | 0.88 | 0.88 | 0.88 | 0.88 | 0.83 |  |
|  | Lower | 0.88 | 0.87 | 0.88 | 0.88 | 0.88 | 0.88 | 0.83 |  |
|  | Upper | 0.88 | 0.87 | 0.88 | 0.88 | 0.88 | 0.88 | 0.83 |  |
|  | F1 Score | 0.67 | 0.67 | 0.67 | 0.65 | 0.69 | 0.68 | 0.58 |  |
